# Perspectives on rigor and reproducibility in single cell genomics

**DOI:** 10.1371/journal.pgen.1010210

**Published:** 2022-05-10

**Authors:** Greg Gibson

**Affiliations:** School of Biological Sciences and Center for Integrative Genomics, Georgia Institute of Technology, Atlanta, Georgia, United States of America; HudsonAlpha Institute for Biotechnology, UNITED STATES

## The promise of single cell genomics

Over the past ten years, single cell genomics has emerged as a powerful approach to cell biology whose impact relative to bulk tissue genomics can be likened to the transition from light to electron microscopy. Query of PubMed with the term “single cell genomics” reveals over 5,000 papers a year since 2012 for a field that is probably in its early growth phase. Analyses at the single cell level reveal properties of tissues that are barely approachable with bulk methods: the contributions of variable cell type abundance to normal and pathological variation, the identities of the cell-types that explain disease, the molecular basis of intercellular signaling, the trajectories of cells as they mature or senesce, and cellular resolution of regulatory mechanisms, are but five examples. None of this would be possible without the parallel emergence of powerful pipelines and algorithms for data analysis, and advances in high performance computing that facilitate routine handling of many-terabyte-sized datasets. Not a week goes by without the publication of a path-breaking study, job-prospects for trainees are outstanding, and fundamental transitions within the “knowledge machine” of modern biology [1] seem inevitable.

Nevertheless, students new to the field can be bewildered by the diversity and complexity of computational methods, and soon realize that single cell data analysis is as much an art as a science. For this reason, there is some room for self-critical evaluation of the existing bioinformatics, and as a contribution to this end I here offer perspectives on five areas where robustness of inference can be improved. The science is going to proceed apace regardless, but I am concerned about a publishing culture that often glosses over the uncertainty that is necessarily intrinsic to analysis of what are among the most complex and voluminous datasets ever produced. We are lucky that the field is building on solid computational and statistical foundations, that attention to experimental quality control is well-informed by recent next-gen sequencing experience, and hence there is good reason to be confident that the vast majority of findings in the literature are correct. Nevertheless, at a time when funding bodies require applicants to address rigor and reproducibility explicitly, it is remarkable how little acknowledgment of analytical variability there is in the literature.

First, though, it is worth emphasizing that there is much to be optimistic about with this young field, starting with the fact that it exists at all. Methods that are commonplace today were not yet even conceived two decades ago when microarray analysis ushered in the era of transcriptomic research, and it is really just a decade since the transition to RNAseq. For a few years it seemed that there were more publications presenting new methods for single cell analysis than experimental findings, but remarkably the field has quickly settled around a few basic pipelines such as Seurat [2], Monocle [3] and Scanpy [4], which is certainly contributing to comparability and accessibility. The current emphasis on generation of comprehensive atlases of single cell profiles for plants and animals [5,6] is a critical step toward understanding the cellular structure of organs and tissues, and will be the foundation for reproducibility. Allied with these efforts is the open sharing of resources, protocols, and code that benefits everyone, particularly as we move rapidly into the era of multimodal and integrative single cell omics. My focus below is on transcriptomics (scRNAseq), but similar considerations apply just as well to single nucleus epigenomic profiling (methylation and ATACseq: [7,8]), spatial sequencing [9,10], single cell proteomics [11] and metabolomics [12], and undoubtedly other soon-to-emerge technologies. Integration of all of these along with 3D imaging, electrophysiology, axon tracing and other methods across species already appears to be the next phase of single cell neuro-biology [13] with similar multimodal approaches promised for other organs.

## Repeatability

To date, the single cell field has failed to establish expectations for reproducibility that are commonplace in other areas of genomics. By way of contrast, much of the confidence in genome-wide association studies might be attributed to rapid establishment of the gold standard that published results both meet the strict genome-wide significance threshold, and show a replicated trend in at least one independent dataset [14,15]. I scanned approximately fifty single cell transcriptomic studies in high impact journals in the past year, and found evidence for external validation in just a handful. Certainly this can reasonably be attributed to the difficulty in obtaining tissues and establishing comparable pipelines, and it is also the case that independent replication in bulk gene expression profiling has not traditionally been required. Large consortia bringing together related single cell datasets is a very positive development, for example the single-cell eQTLGen QTL consortium [16], as it will support both replication and joint analysis while overcoming inevitable constraints governing the design of each contributing study. Resources are generally limited and sources of variability are numerous, so it is important to balance power in discovery studies (perhaps boosted by sample homogeneity) with generalizability in validation work.

In the absence of independently obtained datasets, there are two alternative approaches to enhancing reproducibility that journals could encourage. The first is that investigators endeavor to confirm key findings by an independent team of analysts provided with the same dataset and bioinformatic objective. This is a financial and practical burden beyond the scope of most studies, but high impact research performed by consortia would conceivably benefit from such certification alongside open access to code and datasets that facilitate computational replication by others. It may be that update of MIAME and MINSEQE criteria specifically for annotation of single cell experiments would benefit the community [17]. Whereas GWAS repeatability is high [18] since there are relatively few decision points in the workflow, in my experience the first rule of single cell RNAseq analysis is that two researchers given the same dataset will arrive at different conclusions, often substantially different. The complexity of single cell datasets requires numerous analytical choices and inevitable deviation in outcomes [19,20], and hence independent analytical confirmation can increase confidence. The second approach would be to adopt the principles of cross-validation as a routine component of the bioinformatics. Current practice is typically to draw inference from the entire dataset, rather than to hold out a portion of the samples and validate the conclusions reached from the discovery set. Indeed, in many cases critical findings regarding for example signaling pathways or regulatory mechanisms are drawn from initial partitions of the data, violating the general principle that statistical evaluation not be performed on the discovery data. Thus, an initial analysis may find that cases and controls differ in cell-type A, then differential expression analysis identifies pathway B as the key mediator, whereas a better design is to perform this comparison in an independent sample of cell-type A that may not be subject to the same hidden confounders. Cross-validation approaches [21] have quickly become standard in the polygenic risk assessment field [22], and could be implemented readily in single cell genomics, although I recognize that issues relating to power and balance of partitions need careful attention.

## Clustering

One of the key steps in single cell analysis is the very first post-quality control step of assigning cells to clusters, and this is one of the major sources of irreproducibility. An extraordinary array of tools for clustering has quickly emerged. They are justifiably powering the high resolution of single cell analysis [23], yet new practitioners cannot fail to notice how vexing the process can be. A typical scRNAseq publication proceeds from the identification of some number of clusters that are presented as the ground truth, after which a handful of these are cherry-picked for downstream analysis, sometimes based on the expression of one or a few marker genes of interest. The extent of detail in the description provided in the Methods section is highly variable, and very often insufficient to support replication of the identified clusters by independent investigators [24]. In my group’s experience, it is not unusual for reanalysis to find 20% fewer or more clusters in datasets downloaded from public repositories, with between 50% and 70% equivalence of cell-type assignments. The reasons for this lack of clustering reproducibility are well known, and include analytical decisions made in relation to: QC thresholds, cell-specific normalization, methods used to integrate samples, numbers of highly variable genes and principal components to include, and clustering algorithms. Furthermore, there is little consensus on what evidence is required to identify a set of cells as a distinct cluster: some “splitters” are content with as few as ten differentially expressed transcripts defining a distinct state, whereas “lumpers” err on the side of fewer clusters. Both are legitimate approaches, and it would be inappropriate for reviewers to demand that the authors follow a preferred pipeline strictly: the biologically-aware analyst who works with a dataset day in and day out is in the best position to evaluate the most appropriate approach.

Internal evaluation of cluster reproducibility should be encouraged as a standard aspect in the reporting of single cell studies. Although there is considerable variability across tissue types, my second rule of single cell analysis is that separate partitions of the same dataset, even with the same pipeline, typically result in between 80% and 90% of cells being assigned to the same cluster. For example, if 10% of the cells are removed at random from a dataset, it is common for 10% or more of the remainder to realign to a different cluster when a confusion matrix of assignments is prepared. Similarly, dividing the full dataset into two halves and running the clustering pipeline then comparing replicates of such a partition, will result in a meaningful fraction of cells being reassigned. Or, if the order in which various steps of integration and normalization are performed is altered, cell identities again typically change in a non-trivial manner. In most situations, it is desirable that all clusters be observed in most individuals, and hence permutations based on random removal of samples rather than cells, may be the best strategy for establishing cluster repeatability. These observations argue for community adoption of three standards. (i) Transparent reporting of the criteria used to define clusters and the pipeline used, including finalized code deposition associated with publication as supplementary material. (ii) Reporting of the robustness of cluster assignments in the form of a reproducibility metric, for example a Rand Index, based on one of the above permutation procedures, analogous to the bootstrap support intervals that are commonly included in phylogenetic trees in the molecular evolution literature [25]. (iii) Consideration be given to only including those cells that repeatedly cluster together as the core cells of each cluster for downstream analysis, while those which flip assignments are designated as having ambiguous or unreliable identity.

## Evaluation of significance

It is widely appreciated that hypothesis testing in single cell data is fraught with inconsistency [26,27]. At least three dozen well-validated statistical procedures have been evaluated [28], and while Wilcoxon rank sum tests seem to control both type 1 and type 2 errors reasonably in general, other methods likely have better performance with particular datasets. The problem is that it is never obvious *a priori* which method is most appropriate, and simulation of single cell data to evaluate this is near impossible owing to the variability of zero counts and covariance parameters.

**Fig 1:**
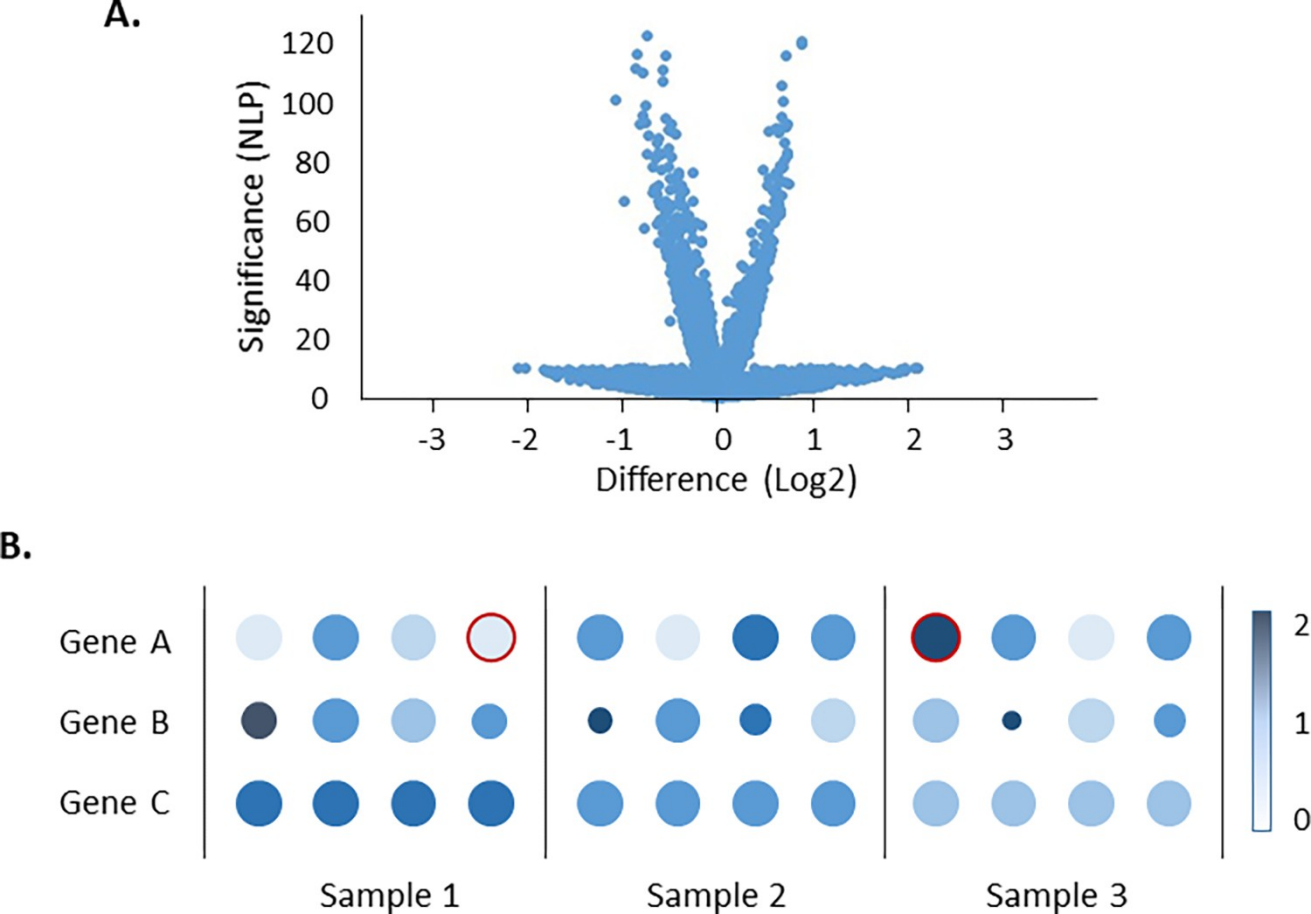
Illustration of Sample Effects. (A) Stylized volcano plot typical of single cell results, showing inflation of test statistics (negative log P-values over 100) and inner and outer bursts of points. (B) Stylized dot plots, with 4 individuals for each of 3 sample types and 3 genes, where color represents intensity of expression (log2 counts per 10,000, scale to right) and size represents proportion of cells. For gene A, Sample 1 appears to have lower expression than the others, but transposition of just the red-outlined individuals between Samples 1 and 3 would equilibrate the pooled expression. For genes B and C, the expression levels are greatest in Sample 1 and least in Sample 3 when pooling all 4 individuals, but given the variability among individuals for Gene B the difference is unlikely to be significant, whereas accounting for individual effects for Gene C enhances the significance since there is low heterogeneity among individuals.

An additional, even more pervasive problem that is largely unrecognized, is the massive misestimation of significance values in single cell data. It is not unusual for differential expression tests to attain p-values in the range of 10^−100^ in comparisons of a half dozen samples that would result in more than an order of magnitude less significance (10^−10^ or less) in bulk RNAseq datasets. Fig 1A stylizes a typical double-volcano plot from a scRNAseq experiment, where the internal bursts of genes have unrealistically high significance for the very small fold changes, and the outer bursts have lower (but still unrealistically high) significance but larger fold changes. The reason for the inflated test statistics is pseudo-replication: cells are treated as if they are biological replicates whereas they should be better regarded as technical replicates. Regardless of the experiment, if a sample is replicated 1000 or more times, extreme significance will result. In the case of the internal bursts, it is uninterpretable, while the external bursts usually result from technical pseudo-replication superimposed on high among-sample variance, likely resulting in a high false positive rate.

Some recent publications have acknowledged this problem [29], but there is as yet no consensus on how to deal with it. My perspective is that confidence in p-values can be misplaced in the context of single cell genomic data. Relative significance is certainly useful, and more informative than simple fold-change measures, but control of false discovery rates and development of optimal discovery procedures [30] in the context of intra-sample correlations that dominate single cell data pose a challenge. A shift to more consideration of variance components would be beneficial. Particularly in the case of complex designs involving two or more fixed effects, such as disease and sex or treatment, for example, as well as interactions among them, it is often of more interest to know which effects have the greatest impact on gene expression, and to know whether the nominally differentially expressed genes are co-regulated and belong to pathways. To this end, applications such as *variancePartition* [31] and Principal Variance Component Analysis [32] that are available for bulk RNAseq data will need to be further developed for appropriate application to single cell datasets.

## Covariate adjustment

Since most single cell genomic datasets derive from relatively small numbers of individual donors, it is common practice for investigators to pool cells from multiple individuals without consideration of the random effect of donor. Worse, the practice of treating each individual sample as a batch is also not infrequent, in which case donor differences are treated as noise to be normalized away, rather than as a source of biological variability that can both control and enhance analyses if treated appropriately as a random effect. The dangers of this are illustrated in Fig 1B, which shows hypothetical dot plots for three genes measured in four individuals of each of three samples. For gene A, five individuals have reduced expression, three in Sample 1, and one each in the other two Samples. Given high levels of technical replication (thousands of cells per individual, often with one individual contributing disproportionate numbers), it would likely be concluded that group 1 has elevated expression if the samples are analyzed as a pool, whereas inclusion of individual as a random effect in a mixed model would reverse the conclusion. The reason is that it is not unlikely that the 3:1:1 ratios of low-expression individuals is observed even if, for example, the true prevalence of the profile is 2:2:1. Conversely, for gene B, which has uneven variability within sample groups, adjustment for individual effects would render the Sample comparison insignificant given the inconsistency of effects, whereas the homogeneity of individual profiles for gene C would render the Sample comparison significant if adjusting for individual as a random effect. These types of influence extend to adjustments for experimental covariates, and only become more important in the presence of interaction effects and heterogeneity of cell numbers across individuals and sample types.

More generally, existing single cell analysis pipelines were not all designed to handle random effects and complex designs that require mixed linear models. Options providing solutions exist, notably MAST [33] and MASC [34]. The former is incorporated into the popular Seurat software as a wrapper, but is most often implemented only to adjust for individual effects. Full linear mixed modeling of single cell data suffers from the additional complication due to the sparsity of data due to the high proportion of zero counts. This sparsity challenges the assumptions of normality of residuals that is the foundation of analysis of variance, and indeed methods based on pseudo-bulk aggregation of cells post-clustering indicate increased power and resolution of multivariate effects in experiments with complex designs [35]. Advances in statistical methodology will come quickly, aiding in optimization of experimental design through tools like scPower [36] and Heirarchicell [37]. These encourage attention to the impact of random individual sample variation and help investigators decide on sequencing depth, cell number and sample size for a fixed budget.

## Normalization

A fifth under-appreciated challenge in single cell transcriptomics, and in fact in gene expression profiling more generally, is the critical role that normalization has for downstream inference [38]. In general, normalization can be categorized with respect to supervised or unsupervised strategies, and with respect to the impact on absolute or relative transcript abundance. Many applications in bulk as well as single cell RNAseq are unsupervised, in the sense that a consistent algorithm is applied to each sample (or cell) without any attempt to adjust for covariates across samples (or cells) that would potentially optimize discovery. Supervised methods implicitly recognize that like samples are expected to have more similar overall profiles, or that technical influences such as RNA quality or true batch effects confound the biological signal, and adjust accordingly [39,40]. Their use is rare in single cell transcriptomics, although tools like *scran* [41] and *PsiNorm* [42] do provide some level of supervision. Instead, the default is to simply convert raw counts to counts per 10,000 reads (typically), perhaps with an adjustment for the influence of the most highly abundant transcripts with *scTransform* [43] for example. There is extensive and important discussion as well around the suitability of treating the data as negative binomially distributed or otherwise [44], but regardless, most single cell methods inherently generate gene expression measures on the relative scale, following the lead introduced when counts per million became the norm with RNAseq. Before then, microarray analysis was typically performed on the absolute scale with normalization by centering of the mean or median to reduce biases due to total amount of RNA.

Whether or not absolute or relative expression is more appropriate is unclear, and may be gene-specific. For some proteins, it matters more that the ratio of product X to product Y shifts from 2:1 to 3:1, whereas for others both products may double in expression with important impacts on cellular physiology despite maintenance of their 2:1 ratio. Transcription factors and kinases may be more likely to be impacted by stoichiometric ratios, whereas enzymes and cytoskeletal proteins may depend on absolute levels, but there are no rules. It follows, however, that detection of differential expression is strongly dependent on mode of normalization. For this reason, it is good practice for analysts to pursue multiple strategies, drawing confidence in robustness where strong agreement is observed, or recognizing potential sources of biological meaning, donor (or mouse or plant, etc) variability, and technical influence where approaches are discordant. Reviewers and editors should be encouraged to promote analytical diversity and regard it as a sign of careful and thoughtful bioinformatics, rather than requiring application of a standard pipeline.

## Conclusion

Single cell genomics is transforming biological research. Widespread access to and adoption of a handful of analytical strategies by a generation of trainees well versed in biocomputing is propelling the field at a rapid pace. On the other hand, training in the underlying statistical foundations is less available, which combined with a culture that favors under-reporting of the effect of analytical decisions, is contributing to over-confidence. Even novice practitioners are well aware of the instability of quantitative findings, yet are being asked to gloss over these in the preparation of tidy analyses that fail to address issues of reproducibility. Twelve years ago, Ioannidis et al [45] published a shocking study of the low repeatability of microarray-based gene expression, in which they failed to computationally replicate, even approximately, more than half of the key findings in 18 high profile studies. The problem in relation to single cell profiling is likely to be at least as great. I hope that implementation of some of the recommendations in this perspective will encourage more biologist-statistician collaboration, and nudge the field toward more acceptance of the ambiguity in single cell genomic interpretation as a consequence of the complexity of the datasets. Uncertainty can be a friend of scientific inference.
